# A nitroxides-based macromolecular MRI contrast agent with an extraordinary longitudinal relaxivity for tumor imaging via clinical T1WI SE sequence

**DOI:** 10.1186/s12951-021-00990-6

**Published:** 2021-08-14

**Authors:** Shiwei Guo, Xiaoming Wang, Zhiqian Li, Dayi Pan, Yan Dai, Yun Ye, Xiaohe Tian, Zhongwei Gu, Qiyong Gong, Hu Zhang, Kui Luo

**Affiliations:** 1 Laboratory of Stem Cell Biology, and Huaxi MR Research Center (HMRRC), Department of Radiology, Frontiers Science Center for Disease-Related Molecular Network, State Key Laboratory of Biotherapy, West China Hospital, National Clinical Research Center for Geriatrics, Sichuan University, 610041 Chengdu, China; 2 Department of Pharmacy of the Affiliated Hospital of Southwest Medical University, Southwest Medical University, Luzhou, 646000 Sichuan People’s Republic of China; 3 Nuclear Medicine and Molecular Imaging Key Laboratory of Sichuan Province, Luzhou, 646000 People’s Republic of China; 4 Functional and Molecular Imaging Key Laboratory of Sichuan Province, Research Unit of Psychoradiology, Chinese Academy of Medical Sciences, Chengdu, 610041 China; 5 Department of Radiology, Chongqing General Hospital, University of Chinese Academy of Sciences (UCAS), No.104 Pipashan Main Street, Yuzhong District, Chongqing, 400014 China; 6 Amgen Bioprocessing Centre, Keck Graduate Institute, Claremont, CA 91711 USA

**Keywords:** Nitroxides-based contrast agents, Longitudinal relaxivity, Magnetic resonance imaging, Cancer diagnosis, Nanomedicines, Polymeric carriers

## Abstract

**Background:**

Macromoleculization of nitroxides has been an effective strategy to improve low relaxivities and poor in vivo stability, however, nitroxides-based metal-free magnetic resonance imaging (MRI) macromolecular contrast agents (mCAs) are still under-performed. These mCAs do not possess a high nitroxides content sufficient for a cumulative effect. Amphiphilic nanostructures in these mCAs are not stable enough for highly efficient protection of nitroxides and do not have adequate molecular flexibility for full contact of the paramagnetic center with the peripheral water molecules. In addition, these mCAs still raise the concerns over biocompatibility and biodegradability due to the presence of macromolecules in these mCAs.

**Results:**

Herein, a water-soluble biodegradable nitroxides-based mCA (Linear pDHPMA-mPEG-Ppa-PROXYL) was prepared via covalent conjugation of a nitroxides (2,2,5,5-tetramethyl-1-pyrrolidinyl-*N*-oxyl, PROXYL) onto an enzyme-sensitive linear di-block poly[*N*-(1, 3-dihydroxypropyl) methacrylamide] (pDHPMA). A high content of PROXYL up to 0.111 mmol/g in Linear pDHPMA-mPEG-Ppa-PROXYL was achieved and a stable nano-sized self-assembled aggregate in an aqueous environment (ca. 23 nm) was formed. Its longitudinal relaxivity (*r*_*1*_ = 0.93 mM^− 1^ s^− 1^) was the highest compared to reported nitroxides-based mCAs. The blood retention time of PROXYL from the prepared mCA in vivo was up to ca. 8 h and great accumulation of the mCA was realized in the tumor site due to its passive targeting ability to tumors. Thus, Linear pDHPMA-mPEG-Ppa-PROXYL could provide a clearly detectable MRI enhancement at the tumor site of mice via the T1WI SE sequence conventionally used in clinical Gd^3+^-based contrast agents, although it cannot be compared with DTPA-Gd in the longitudinal relaxivity and the continuous enhancement time at the tumor site of mice. Additionally, it was demonstrated to have great biosafety, hemocompatibility and biocompatibility.

**Conclusions:**

Therefore, Linear pDHPMA-mPEG-Ppa-PROXYL could be a potential candidate as a substitute of metal-based MRI CAs for clinical application.

**Graphic Abstract:**

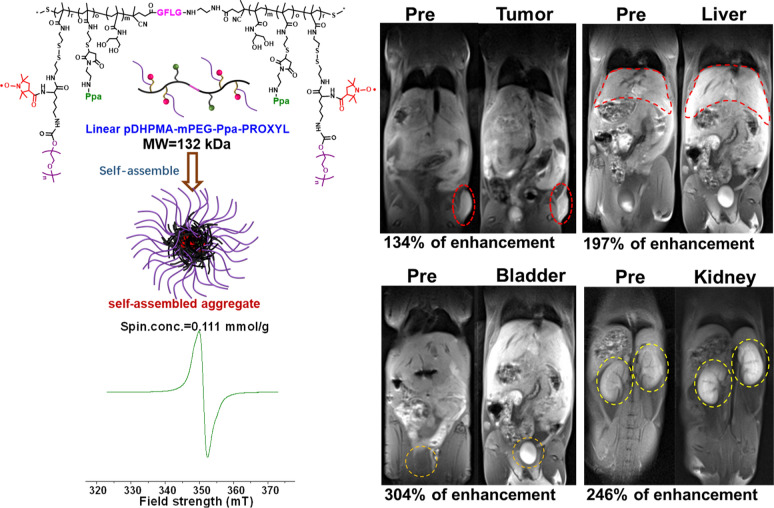

**Supplementary Information:**

The online version contains supplementary material available at 10.1186/s12951-021-00990-6.

## Introduction

Contrast agents (CAs) play an essential role in obtaining accurate diagnosis results for the current magnetic resonance imaging (MRI) technology [1–4]. The majority of MRI CAs are derived from paramagnetic (Gd^3+^ and Mn^2+^) or superparamagnetic (Fe_3_O_4_) metallic materials [5–10]. However, metal-associated toxicity has raised concerns over the use of these metal-based CAs [11]. Therefore, it is of great significance to develop metal-free CAs that have a high biosafety profile and meet the requirements of clinical diagnosis application [12]. Nitroxides are unalloyed organic compounds containing single spin electrons [13, 14]. Due to their paramagnetism, nitroxides can affect the longitudinal relaxation time (T1) of water protons, thus creating relaxivity, which shares the same mechanism as most commonly used Gd^3+^-based CAs [15–19]. Among nitroxides, 2,2,5,5-tetramethyl-1-pyrrolidinyl-*N*-oxyl (PROXYL) and 2,2,6,6-tetramethylpiperidinyl-1-oxyl (TEMPO) are mostly studied because of their relatively high stability [20–23]. However, in order to realize clinical application of nitroxides, two major hurdles should be overcome: their poor stability in vivo due to their high sensitivity to reducing substances in the body (such as glutathione) and low relaxivities due to their inherent magnetic properties [24–29].

Previous studies have shown that the in vivo stability and relaxivities of nitroxides can be improved by loading them on macromolecular materials to construct metal-free macromolecular CAs (mCAs) [22, 30–34]. Inspired by their encouraging research outcomes, we recently introduced small molecular PROXYL into linear and cross-linked PEGylated polyesters to construct two water-soluble metal-free mCAs (linear and cross-linked PCE-mPEG-Ppa-PROXYL) [35]. Compared with the linear counterpart, cross-linked PCE-mPEG-Ppa-PROXYL had a more rigid amphiphilic structure, and more stable nano-scale self-aggregates were formed in a physiological environment, which could not only improve the relaxivity of PROXYL via the macromolecular effect, but also contributed to protecting PROXYL in the hydrophobic core of the aggregate. At the same time, the cross-linked mCA had enough structural flexibility of allowing sufficient contact of the paramagnetic center with its surrounding water molecules, which was conducive to improving the relaxation efficiency. As a result, the cross-linked PCE-mPEG-Ppa-PROXYL provided a better MR imaging effect in vivo than the linear analogue. In addition, great biodegradability of the polyester main chain in the mCA reduces the toxicity risks derived from macromolecules [35]. Although this mCA has outperformed other previously reported metal-free mCAs, the contrast enhancement effect from the cross-linked PCE-mPEG-Ppa-PROXYL was only highly sensitive when the T_1_ mapping sequence was used, while the MR signal contrast became insensitive for the T1WI SE sequence. The results could be due to a low relaxivity of this mCA owing to the inadequate content of nitroxides in the mCA because an increase in the nitroxides content in this mCA led to a decrease in its water solubility.

From our recent study and other reported studies, ideal nitroxides-based mCAs should meet the following requirements simultaneously: (1) they are amphiphilic to self-assemble into stable nano-sized structures; (2) the content of nitroxides in mCAs should be high enough for enhancing the imaging contrast; (3) they should have structural flexibility for rapid energy exchange and sufficient contact between the paramagnetic center and the surrounding water molecules; and (4) they should have excellent biodegradability to reduce toxicity of macromolecules in mCAs [10, 17, 25, 35, 36].

Linear biodegradable block poly[*N*-(1, 3-dihydroxypropyl) methacrylamide] (pDHPMA) with bio-cleavable components in its main chain is an excellent macromolecular carrier that has been used for delivery of drugs and imaging probes [37–39]. These linear biodegradable block DHPMA copolymers as a delivery carrier for nitroxides have their unique advantages over other macromolecules. They could be easily prepared and their structures could be precisely tuned for MRI application. A high density of functional groups in DHPMA copolymers endows them with a high load capacity for nitroxides. After precise regulation of the ratio of hydrophobicity and hydrophilicity in DHPMA copolymers, they could self-assemble into stable aggregates at nano-meter sizes. In addition, they have moderate molecular flexibility and excellent biocompatibility and biodegradability [37–39]. Therefore, linear biodegradable block DHPMA copolymers were chosen for constructing nitroxides-based metal-free mCAs in this study.

**Fig. 1:**
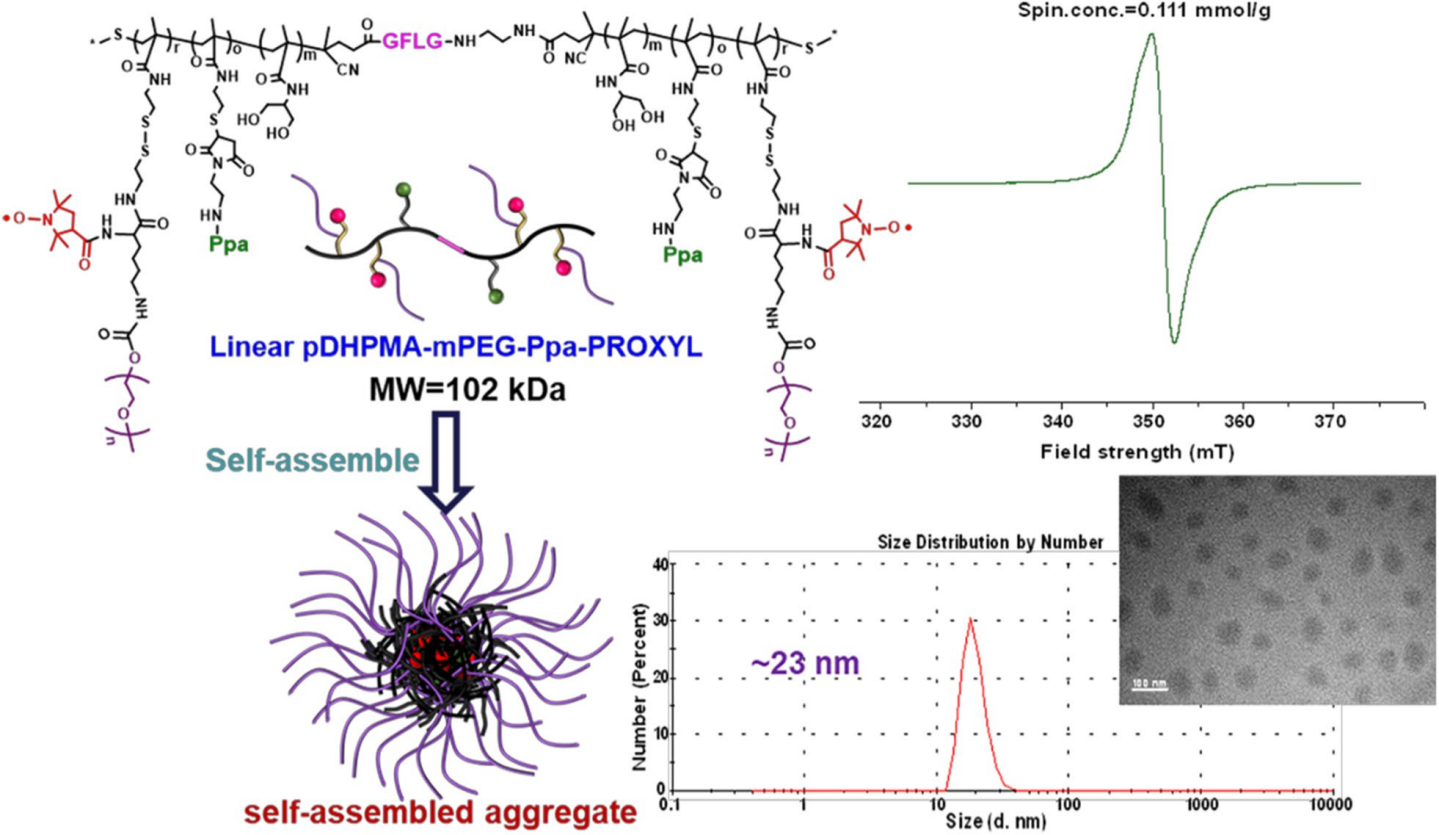
Chemical structure and properties of Linear pDHPMA-mPEG-Ppa-PROXYL

A linear di-block DHPMA copolymer (Linear pDHPMA-SH) was prepared, which was interlinked through enzyme-sensitive short peptides (Gly-Phe-Leu-Gly, GFLG) and equipped with rich thiols in the side chain [37]. A great number of PEGylated PROXYL derivatives (PTE-mPEG-PROXYL) were covalently conjugated onto the Linear pDHPMA-SH via efficient thiol-disulfide exchange reaction, and a trace amount of a fluorescent agent (pheophorbide-a, Ppa) was introduced for intracellular distribution analysis, resulting in a biodegradable PROXYL-based metal-free mCA (Linear pDHPMA-mPEG-Ppa-PROXYL) (Fig. 1). The mCA could self-assemble into a nanostructure to wrap PROXYL in the hydrophobic core within a hydrophilic layer so that PROXYL could be protected during blood circulation. The nanostructure could passively accumulate at the tumor site for enhancing the imaging effect. After in-vitro evaluation of the longitudinal relaxivity and cytotoxicity/hemocompatibility of this mCA, this mCA was also injected into tumor-bearing mice for MR imaging of major organs and tumors using the T1WI SE sequence conventionally used in clinical Gd^3+^-based contrast agents. Clearly detectable MRI signal enhancements and excellent biosafety properties of this metal-free mCA could pave a way for its clinical application.

## Materials and methods

Preparation and characterizations of Linear pDHPMA-mPEG-Ppa-PROXYL were detailed in Additional file 1. Additionally, experiments including toxicity evaluation in vivo and in vitro, blood compatibility and cellular uptake were also included in the Supporting Information.

### Animal models

All experimental BALB/c mice (8–10 weeks, 20 ± 2 g) were purchased from Chengdu Daso Experimental Animals Co., LTD. 4T1 cells from mouse breast cancer cell lines were used to establish a mouse breast cancer model in mice. 4T1 cells were inoculated into the fourth pair of mammary glands of mice at a density of 7 × 10^5^. When the tumor diameter reached 100 mm^3^, these mice were ready for MR imaging.

### MR imaging

The longitudinal relaxivity of Linear pDHPMA-mPEG-Ppa-PROXYL in phosphate buffer saline (PBS) was measured using a clinical Siemens 3.0-T MR imaging unit (Trio Tim). Linear pDHPMA-mPEG-Ppa-PROXYL at different concentrations of PROXYL (0, 0.17, 0.34, 0.68, 1.35, 2.7, 5.4, 10.8 mM) was dissolved in 0.1 M PBS, and MR signals of the prepared solutions were scanned by T1 SE sequence. Image acquisition parameters were set as an echo time of 6.9 ms, a repetition time of 20, 30, 50, 70, 90, 125, 150, 175, 200, 300, 400, 500, 700, 850, and 1000 ms, a Fov of 200 × 200 mm, a slice thickness of 1.0 mm. The corresponding 1/T1 values were obtained from their T1-weighted MR images. The value of relaxivity (*r*_1_) was calculated by plotting 1/T1 as a function of different PROXYL concentrations. In addition, a small molecular PROXYL compound (3-Carboxy-PROXYL, 3-CP) at the same concentration in 0.1 M PBS was used as a control.

Twenty 8–10-week healthy female BALB/c mice were randomly divided into four groups (5 in each group, 20 ± 2 g). Another ten female BALB/c tumor-bearing mice (20 ± 2 g, 8–10 weeks) were also randomly divided into two groups (n = 5). The MR signals of main organs including the liver, kidney and bladder and tumors in the body at different time points were obtained via a clinical Siemens 3.0-T MR imaging unit (Trio Tim). The mice were anesthetized with 1–2% isoflurane and fixed in the magnetic resonance radio frequency coil (Shanghai Chenguang Medical Technology Co., Ltd., model: CG MUC23 H300 AS with an eight-channel phased array structure). The T1WI SE sequence was used for coronal scanning. Image acquisition parameters were set as a repetition time of 500 ms, an echo time of 11 ms, a field of view of 78 × 78 mm, a voxel size of 0.3 × 0.3 × 1.2 mm^3^, a section thickness of 1.2 mm, a slice space of 2 mm, and 14 image sections. Scanning was executed at pre-injection, 5, 10, 15, 20, 25, and 30 min after injection of Linear pDHPMA-mPEG-Ppa-PROXYL and 3-CP at an equivalent dose of 0.135 mmol/kg PROXYL to obtain images of each organ and tumor by MR imaging physicists, and the enhancement (SI%) in the MRI signal intensity (SI) was obtained in the same approach as previously reported [28, 35].

### In vivo metabolism

Ten female healthy BALB/c mice (8–10 weeks, 20 ± 2 g) were randomly divided into two groups (n = 5), and Linear pDHPMA-mPEG-Ppa-PROXYL and 3-CP were injected through the tail vein at a dose of 0.135 mmol/kg PROXYL, respectively. 50 µL of blood samples were collected through the fundus vein at time points of 3 min, 6 min, 9 min, 12 min, 15 min, 30 min, 1 h, 2 h, 4 h, 8 h, and 24 h after injection. The blood samples were centrifuged (10,000*g* × 5 min) and the serum supernatants were collected. The spin concentration (the nitroxides content) of each serum sample at different time points was measured by electronic paramagnetic resonance (EPR).

## Results and discussion

### Preparation and characterizations of Linear pDHPMA-mPEG-Ppa-PROXYL

Biodegradable block DHPMA copolymers have a precisely adjustable structure and plenty of functional groups for covalent binding, therefore, these pDHPMA-based drug delivery systems can form water-soluble stable morphologies at different nano-scale sizes with a high content of active components. Thus, DHPMA copolymers were chosen as macromolecular carriers for construction of nitroxides-based mCAs. Since the macromolecular structure should have good flexibility to allow the paramagnetic center to completely and rapidly interact with surrounding water molecules, in this study, a thiols-functionalized linear biodegradable di-block DHPMA copolymer with enzyme-sensitive GFLG peptides in the main chain (Linear pDHPMA-SH) was prepared to develop a novel nitroxides-based mCA (Linear pDHPMA-mPEG-Ppa-PROXYL, Additional file 1: Scheme S1).

We first prepared a linear DHPMA copolymer via reversible addition-fragmentation chain transfer (RAFT) polymerization of a water-soluble monomer (DHPMA), a pyridinyldithiol (PTE)-functionalized monomer (PTEMA) and a chain transfer agent (CTA-GFLG-NH-CTA) induced by VA044. Pyridyl-thiols in the PTE groups were removed via reduction to obtain thiol-functionalized Linear pDHPMA-SH (Additional file 1: Scheme S1) whose main chain could be specifically bio-cleaved by cathepsin B highly expressed in tumor cells. Additionally, a dithiopyridyl-functionalized PROXYL derivative containing methyl polyethylene glycol chain (mPEG) with a molecular weight of 2 kDa (PTE-mPEG-PROXYL) and a maleimide-functionalized fluorescent probe (pyropheophorbide-α) derivative (Ppa-Maleimide) were also prepared according to previously reported methods [37, 40]. PTE-mPEG-PROXYL and Ppa-Maleimide were covalently bound onto Linear pDHPMA-SH via thiol-disulfide exchange reaction and thiol-ene click chemistry, respectively, resulting in Linear pDHPMA-mPEG-Ppa-PROXYL (Additional file 1: Scheme S1). In the synthesis process of the DHPMA copolymer, we adjusted the ratio of monomers to achieve a high water-solubility and a high loading capacity. The monomer DHPMA contributed to the water solubility of the mCA, while an increase in the proportion of the functional monomer (PTEMA) could lead to a high content of PROXYL in the mCA. The PTE-functionalized PEGylated PROXYL derivative (PTE-mPEG-PROXYL) was introduced onto the mCA to balance its water solubility and a high load of PROXYL. Moreover, the introduction of PEG chains could increase the molecular weight of the mCA, thus enhancing the macromolecular effect. In addition, PEGylation also helps to reduce immunogenicity, enhance biocompatibility, prolong a half-life of mCAs [41, 42]. The amount of Ppa-Maleimide in the mCA was controlled to be below 1% wt, which was adequate for fluorescence imaging but did not affect the water solubility of the final contrast agent [35].

Chemical properties of the copolymer and mCA were listed in Additional file 1: Table S1. The chemical structure of Linear pDHPMA-mPEG-Ppa-PROXYL was determined by comparing ^1^H NMR spectra (Additional file 1: Fig. S1). MWs (102 kDa vs 56 kDa, Additional file 1: Table S1) of the copolymer pDHPMA-SH were determined before and after reaction with PTE-mPEG-PROXYL and Ppa-Maleimide. According to the EPR analysis, this mCA displayed strong paramagnetism and its spin concentration or the PROXYL content was 0.111 mmol/g (Additional file 1: Fig. S2). In addition, dynamic light scattering (DLS) and transmission electron microscope (TEM) analysis results revealed that Linear pDHPMA-mPEG-Ppa-PROXYL formed stable nano-sized self-assembled aggregates (ca. 23 nm) in an aqueous solution due to a tuned balance between hydrophilic and hydrophobic components in the mCA (Additional file 1: Figs. S3, S4). The nano-sized aggregate structure could effectively protect PROXYL distributed in the hydrophobic core during blood circulation by the hydrophilic periphery, and a nanoscale size also endowed the mCA with a passive targeting ability to tumors [43]. The presence of GFLG peptides in Linear pDHPMA-mPEG-Ppa-PROXYL was confirmed from amino acid analysis. The content ratio of Gly, Phe, Leu and Lys was determined to be ca. 1.60/1.91/1.15/1 (Additional file 1: Table S2), which matched the theoretical ratio of these three amino acids in the GFLG peptide. Additionally, the zeta potential of Linear pDHPMA-mPEG-Ppa-PROXYL was ca. 0 mV from DLS analysis (Additional file 1: Fig. S5), which could help reducing adsorption of charged proteins in the blood onto the mCA and prolonging its blood circulation time.

### Relaxivity of Linear pDHPMA-mPEG-Ppa-PROXYL

**Fig. 2:**
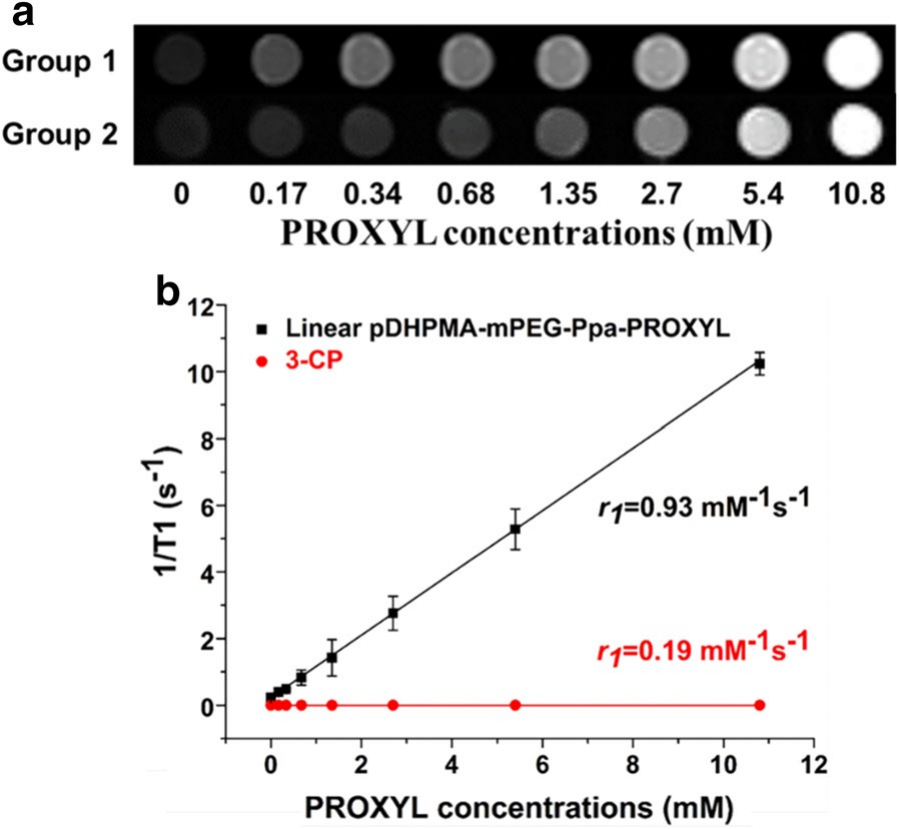
**a** MRI signals of Linear pDHPMA-mPEG-Ppa-PROXYL (Group 1) and 3-CP (Group 2) and **b** their derived longitudinal relaxivities (*r*_1_) in vitro

Evaluation of the relaxivity (*r*_1_) of Linear pDHPMA-mPEG-Ppa-PROXYL was conducted by a clinical Siemens 3.0 T MRI scanner, and 3-CP was used as a control group. As shown in Fig. 2a, at the equivalent PROXYL concentration, stronger signals were seen from the Linear pDHPMA-mPEG-Ppa-PROXYL samples than 3-CP in their MRI images. The in vitro relaxivity of Linear pDHPMA-mPEG-Ppa-PROXYL (*r*_*1*_ = 0.93 mM^− 1^ s^− 1^) was significantly higher than that of 3-CP (*r*_1_ = 0.19 mM^− 1^ s^− 1^) (Fig. 2b). A unique structural design in Linear pDHPMA-mPEG-Ppa-PROXYL contributed to its higher relaxivity. A macromolecular structure (pDHPMA) for the MRI contrast agent substantially increased the rotation correlation time (τ_R_). In addition, Linear pDHPMA-mPEG-Ppa-PROXYL had a high content of nitroxides, which increased the energy exchange with surrounding water molecules. At the same time, the self-assembled aggregate had great flexibility for complete contact of nitroxides and their surrounding water molecules, resulting in rapid energy transfer between their single spin electrons. However, in the control group, the molecular weight of 3-CP is much smaller and its molecular roll-over speed is faster, which leads to a decrease in its τ_R_. According to the Bloembergen–Solomon–Morgan theory, a lowered τ_R_ results in a reduced relaxivity.

Furthermore, compared to the mCA prepared from ched-dendrimers (*r*_1_ = 0.42 mM^− 1^ s^− 1^) by the Rajca research group [28] and the mCA prepared from cross-linked carboxylate ester (*r*_1_ = 0.79 mM^− 1^ s^− 1^) from our previous study [35], Linear pDHPMA-mPEG-Ppa-PROXYL had a higher *r*_1_ value (0.93 mM^− 1^ s^− 1^). Although the spin concentration of the ched-dendrimer-based mCA (0.41 mmol/g) and cross-linked carboxylate ester-based mCA (0.135 mmol/g) was higher than that of Linear pDHPMA-mPEG-Ppa-PROXYL (0.111 mmol/g), the molecular weight of Linear pDHPMA-mPEG-Ppa-PROXYL (102 kDa) was significantly larger than that of ched-dendrimer-based mCA (32 kDa) and cross-linked carboxylate ester-based mCA (26.1 KDa), so Linear pDHPMA-mPEG-Ppa-PROXYL had a stronger macromolecular effect. In addition, compared with rigid dendrimers and cross-linked carboxylate esters, DHPMA copolymers were highly flexible, and the paramagnetic center in Linear pDHPMA-mPEG-Ppa-PROXYL could quickly contact with peripheral water molecules and exchange energy between them. Therefore, the longitudinal relaxivity of Linear pDHPMA-mPEG-Ppa-PROXYL was higher than that of the mCA prepared from ched-dendrimers and cross-linked carboxylate esters.

### In vivo blood circulation of PROXYL

**Fig. 3:**
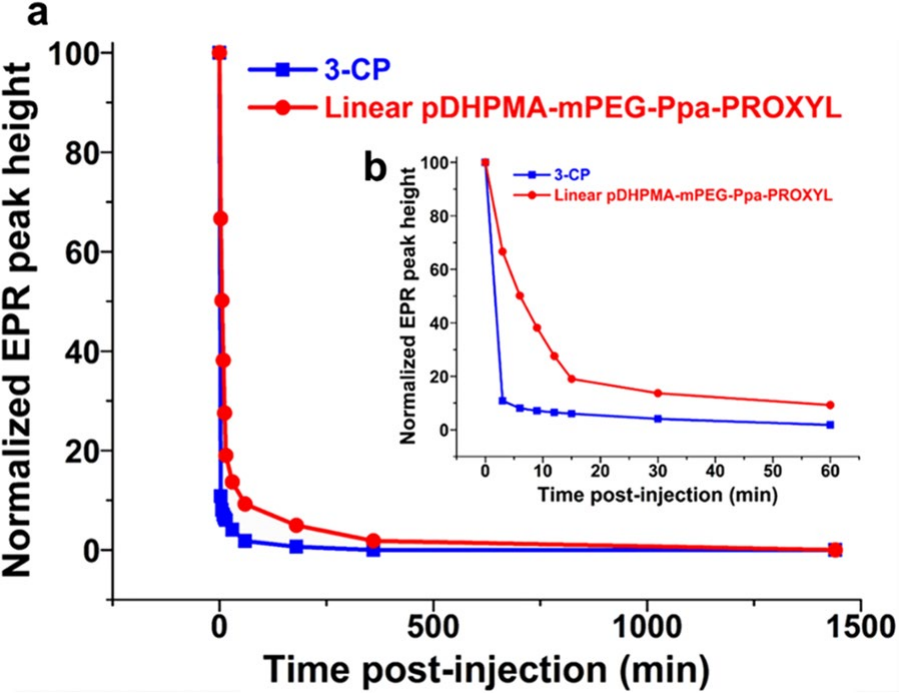
**a** Temporary concentration changes of nitroxides after injection of Linear pDHPMA-mPEG-Ppa-PROXYL and 3-CP in normal mice for 24 h. **b** The concentration changes within 1 h shown in the inset plot

As shown in Fig. 3, the PROXYL concentration in the 3-CP group in blood samples decreased rapidly and it was undetectable within 1 h, however, although the PROXYL concentration in the Linear pDHPMA-mPEG-Ppa-PROXYL group had a sharp reduction in the first 5 min, the reduction gradually slowed down and PROXYL was still detectable up to 8 h after injection. The macromolecular structure of Linear pDHPMA-mPEG-Ppa-PROXYL played a critical role in extending the circulation time of nitroxides in the blood. First, the nitroxides were protected in the hydrophobic core of self-assembled aggregates, reducing the interaction between endogenous reducing substances in the blood and nitroxides embedded in Linear pDHPMA-mPEG-Ppa-PROXYL, thereby, extending the detection duration for nitroxides residues in the blood up to 8 h post-injection. Moreover, the macromolecular structure in Linear pDHPMA-mPEG-Ppa-PROXYL could be slowly metabolized and the mCA could maintain its structure for a relatively long time in the blood.

### In vivo MR imaging of major organs

**Fig. 4:**
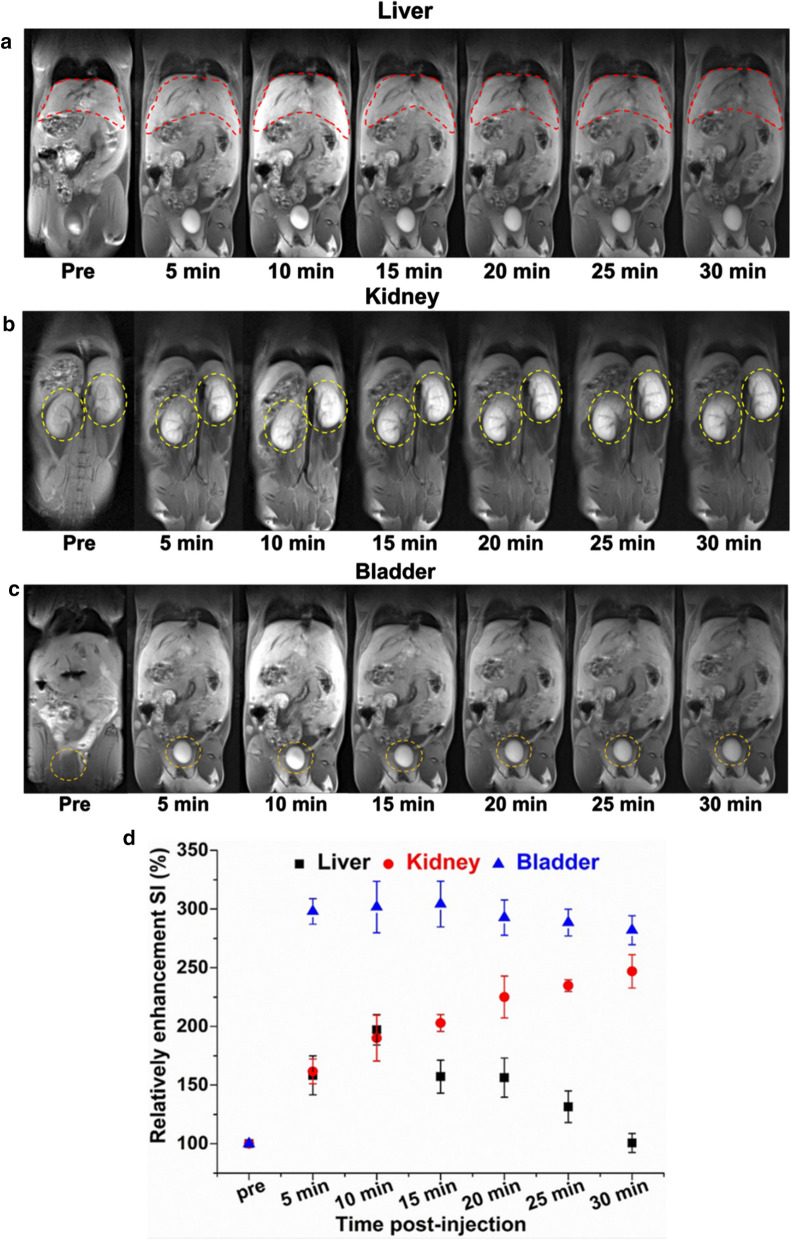
Representative MR T1-weighted images of the liver (**a**), kidney (**b**) and bladder (**c**) of one mouse after injection of Linear pDHPMA-mPEG-Ppa-PROXYL. **d** Quantitative analysis of the relative enhanced signal ratios in the liver, kidney, and bladder after injection of Linear pDHPMA-mPEG-Ppa-PROXYL

MR imaging of major organs including the liver, kidney and bladder of healthy BALB/c mice were conducted after injection of Linear pDHPMA-mPEG-Ppa-PROXYL and 3-CP. Analysis of signal intensity (SI) in these MR images revealed that the SI in the liver and bladder reached a peak of signal enhancement at 10 min, and the degrees of enhancement were about 197 and 304% in the liver and bladder, respectively (Fig. 4d). After 30 min, the enhancement in the liver reduced to the baseline (Fig. 4a), while significant signal enhancement by the mCA was evident in the bladder (Fig. 4b). In the kidney (Fig. 4c), gradual increase in the signal intensity was observed within 5–30 min. The degree of signal enhancement increased about 246% at 30 min compared with that at pre-injection (Fig. 4d). However, after injection of 3-CP as a control group, there was no MRI enhancement in all major organs (Additional file 1: Fig. S6). Therefore, injection of Linear pDHPMA-mPEG-Ppa-PROXYL to mice resulted in obvious enhancement in the MR imaging in the liver and kidney, with sharp tissue contrast and a long imaging window time. Incremental signal intensity in the kidney may suggest that Linear pDHPMA-mPEG-Ppa-PROXYL could be metabolized by the kidney and eliminated from the body, which could ensure biosafety of the contrast agent. At the same time, the T1WI SE sequence was applied to detection of MRI signals generated from Linear pDHPMA-mPEG-Ppa-PROXYL and the degrees of MR signal enhancement were achieved in major organs, which could pave a way forward its clinical application.

### In vivo MR imaging of tumors

**Fig. 5:**
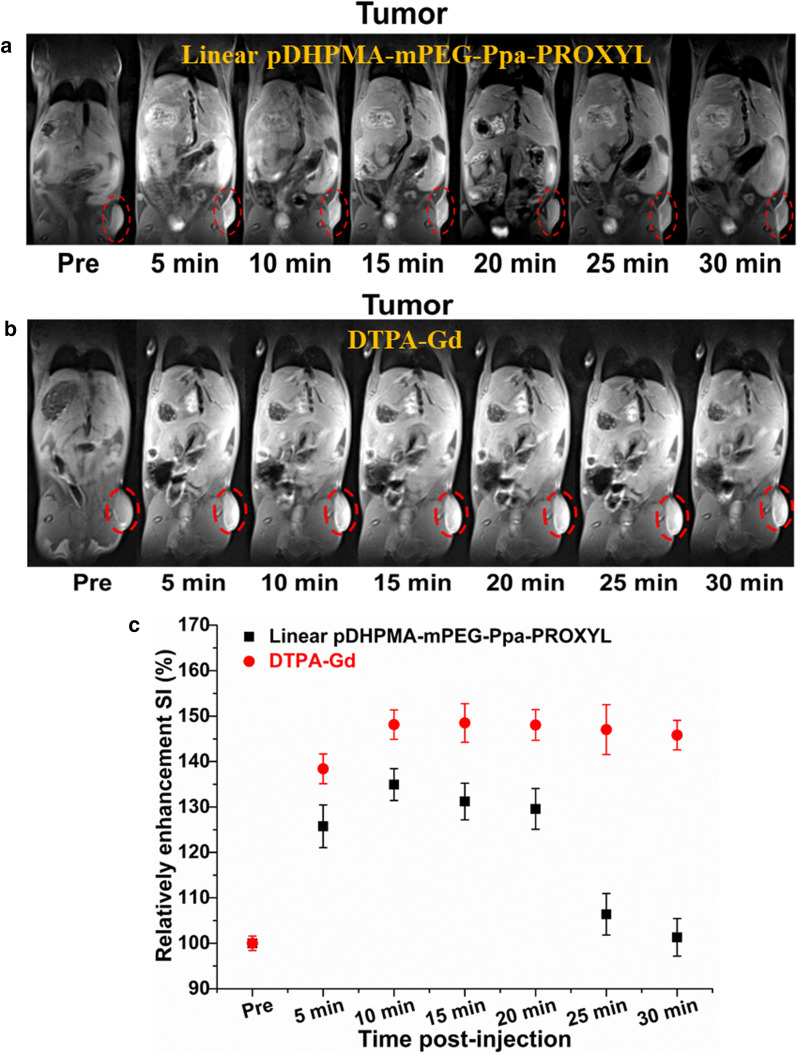
Representative MR T1-weighted images of tumors of one mouse after injection of Linear pDHPMA-mPEG-Ppa-PROXYL (**a**) or DTPA-Gd (**b**). **c** Quantitative analysis of the relative enhanced signal ratio at the tumor site after injection of Linear pDHPMA-mPEG-Ppa-PROXYL or DTPA-Gd

Since reducing substances (such as glutathione) in the tumor site have a high concentration and they can readily convert paramagnetic nitroxides into non-magnetic hydroxylamine compounds, nitroxides-based MR CAs often rapidly lose their relaxation effect, resulting in inadequate imaging contrast and poor structural stability at the tumor site. We have demonstrated that Linear pDHPMA-mPEG-Ppa-PROXYL possessed a higher relaxivity in vitro and longer-term stability in vivo. Therefore, we further evaluated the imaging effect of Linear pDHPMA-mPEG-Ppa-PROXYL at tumor sites in vivo with 3-CP and clinical Gd-based CA (DTPA-Gd) as controls. As shown in Fig. 5a, after administration of Linear pDHPMA-mPEG-Ppa-PROXYL, the MRI SI at the tumor site was enhanced within 20 min, reaching the maximum of about 134% at 10 min post injection. Afterwards, it started to decrease down to the original baseline at 30 min (Fig. 5c). In the DTPA-Gd-injected group (Fig. 5b), MR signal at the tumor site was gradually enhanced within 30 min after injection, with a maximum enhancement peak of about 150% (Fig. 5c). The mice injected with small molecular 3-CP were scanned by the same method, but no enhancement in the MR signal at the tumor site was found (Additional file 1: Fig. S7). It could be seen that the enhancement effect by Linear pDHPMA-mPEG-Ppa-PROXYL at the tumor site, displaying strong imaging contrast and a prolonged imaging duration. Although it underperformed in comparison with DTPA-Gd, as a nitroxides-based mCA, the enhanced signal at the tumor site can be found by the T1WI SE sequence conventionally used in clinical Gd^3+^-based CAs, such result is also encouraging. Both the imaging contrast and imaging duration achieved by Linear pDHPMA-mPEG-Ppa-PROXYL were better than those from previously reported nitroxides-based mCAs [24–28, 35]. Such an excellent imaging effect could be explained by a high content of PROXYL in Linear pDHPMA-mPEG-Ppa-PROXYL, passive accumulation at tumor sites, and protection of PROXYL in the amphiphilic nanostructures. Meanwhile, the change in the MRI enhanced signal were detected by the T1WI SE sequence, which could help readily translating this mCA as an MRI contrast agent into clinical use.

### In vitro uptake of the contrast agent by 4T1 cells

**Fig. 6:**
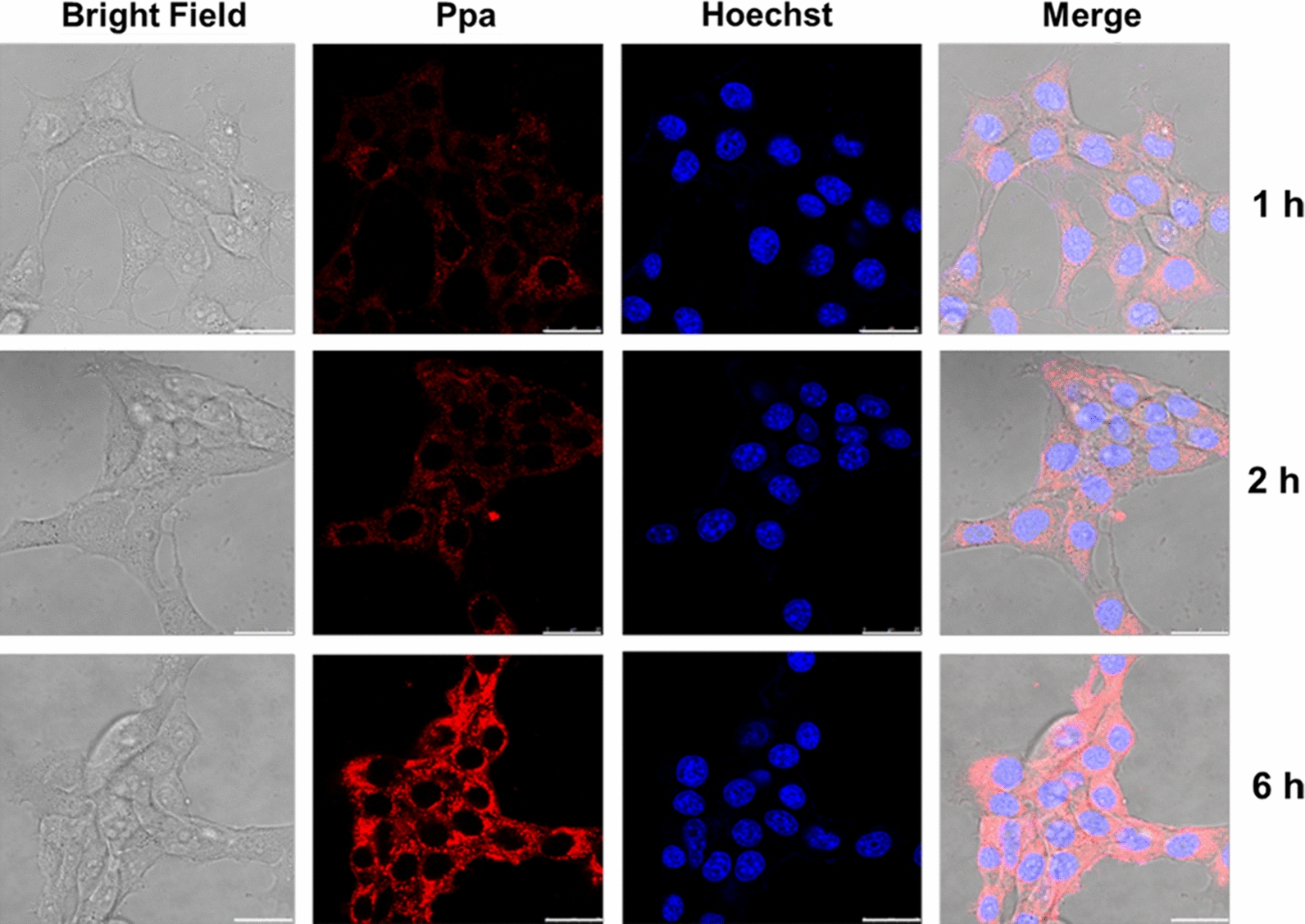
Fluorescence images of 4T1 cells incubated with Linear pDHPMA-mPEG-Ppa-PROXYL for 1 h, 2 h, and 6 h. Linear pDHPMA-mPEG-Ppa-PROXYL (red fluorescence) was completely distributed in the cytoplasm without entering the nucleus (blue fluorescence), and the cellular uptake of this mORCA by 4T1 cells was a time-dependent process. Scale bar: 25 μm

As shown in Fig. 6, weak red fluorescence of Linear pDHPMA-mPEG-Ppa-PROXYL in the cytoplasm of 4T1 cells was noticeable at 1 h, and the cytoplasmic fluorescence intensity was significantly intensified at 6 h compared to that at first 2 h. Linear pDHPMA-mPEG-Ppa-PROXYL completely accumulated in the cytoplasm and did not enter the nucleus since red fluorescence did not overlap with blue fluorescence in these CLSM images for up to 6 h. The experimental results supported that Linear pDHPMA-mPEG-Ppa-PROXYL could be endocytosed by 4T1 cells into the cytoplasm in a time-dependent manner.

### In vitro cell toxicity

**Fig. 7:**
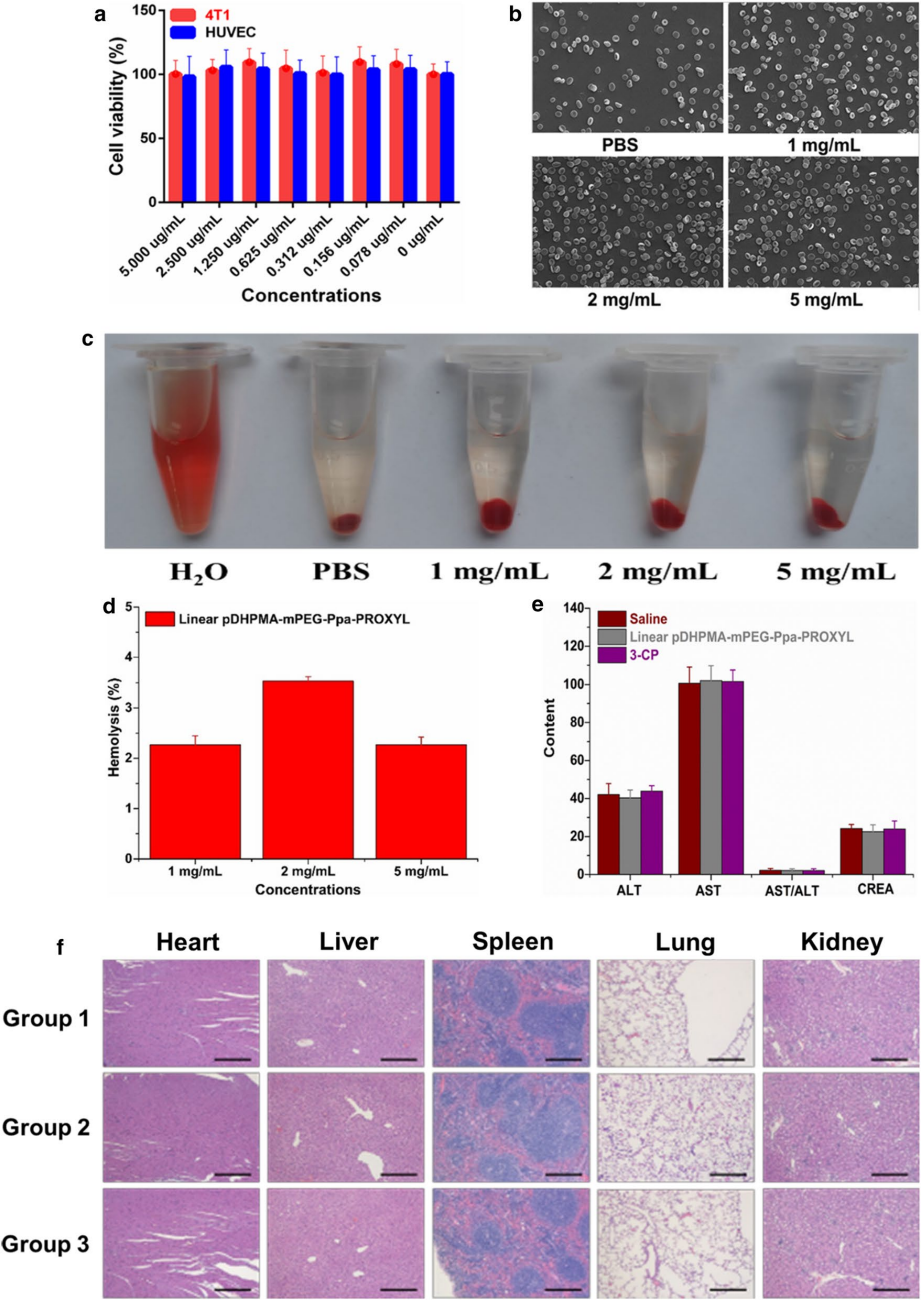
**a** Viabilities of 4T1 cells and HUVEC cells after incubation with Linear pDHPMA-mPEG-Ppa-PROXYL at various concentrations for 24 h. **b** Erythrocyte aggregates and morphologies in PBS or Linear pDHPMA-mPEG-Ppa-PROXYL solutions at 1, 2, and 5 mg/mL. **c** Red blood cell hemolysis induced by Linear pDHPMA-mPEG-Ppa-PROXYL. **d** Hemolysis rates induced by Linear pDHPMA-mPEG-Ppa-PROXYL at different concentrations. **e** Blood chemistry indexes in mice injected with saline, Linear pDHPMA-mPEG-Ppa-PROXYL and 3-CP for 24 h. **f** Histological images of mice 1 day after injection of saline (Group 1), 3-CP (Group 2) and Linear pDHPMA-mPEG-Ppa-PROXYL (Group 3). Scale bar: 10 μm

Cytotoxicity analysis against tumor and normal cells provides great insights into biocompatibility of this MRI mCA. The activity of 4T1 and HUVEC cells was greater than 95 % after 24 h of incubation with Linear pDHPMA-mPEG-Ppa-PROXYL at concentrations ranging from 0 to 5 µg/mL, and there was no significant difference in the activity between 4T1 and HUVEC cells (Fig. 7a). The result indicated that Linear pDHPMA-mPEG-Ppa-PROXYL displayed no obvious toxicity to normal and tumor cells. A neutral surface charge and great biodegradability of Linear pDHPMA-mPEG-Ppa-PROXYL could be ascribed to its excellent cytocompatibility.

### In vitro RBC hemolysis and morphology tests of the contrast agent

After 24 h of incubation, Linear pDHPMA-mPEG-Ppa-PROXYL did not induce any hemolysis in the blood (Fig. 7c). Semi-quantitative analysis via a UV spectrophotometer showed that the hemolysis rate after incubation with the contrast agent at a concentration up to 5 mg/mL was less than 5% (Fig. 7d). In addition, compared with RBCs in PBS, these cells appeared normal after exposure to the contrast agent under a SEM, displaying a double concave disc-like structure without collapse or rupture in the cellular morphologies (Fig. 7b).

### In vivo toxicity of the contrast agent

Three groups of healthy female BALB/c mice (n = 5) were injected with Linear pDHPMA-mPEG-Ppa-PROXYL and 3-CP at 0.135 mmol PROXYL/kg mice through tail vein, and observed for evaluating its toxicity in vivo. Saline was used as a control. After 24 h, the mice did not show acute side effects such as hemorrhage and death. Subsequently, the mice were killed. Main organs were dissected for histological analysis and blood samples were collected for blood chemistry index analysis including aspartate aminotransferase (AST), alanine aminotransferase (ALT) and creatinine (CREA). As shown in Fig. 7, compared with mice injected with 3-CP and saline, the Linear pDHPMA-mPEG-Ppa-PROXYL-injected group had similar blood chemistry indexes (Fig. 7e), and did not show tissue damage and histopathological abnormalities (Fig. 7f). The results supported that Linear pDHPMA-mPEG-Ppa-PROXYL was non-toxic to organs and tissues in vivo at the dose of MR imaging. This could be ascribed to the molecular structure and chemical/physical/biological properties of the mCA, including a high water-solubility, non-immunogenicity, biodegradability of the polymer, and a neutral charge on the polymeric surface [44, 45].

## Conclusions

We have used a thiols-functionalized linear di-block DHPMA copolymer with enzyme-sensitive GFLG peptides in its main chain as a macromolecular skeleton for covalent conjugation with PEGylated PROXYL derivatives, resulting in a novel metal-free mCA with a MW of 102 kDa (Linear pDHPMA-mPEG-Ppa-PROXYL). It had a high longitudinal relaxivity (*r*_1_ = 0.93 mM^− 1^ s^− 1^) due to a high nitroxides content (0.111 mmol/g). In vivo stability of PROXYL in this mCA was significantly enhanced with up to around 8 h of the blood retention time due to efficient protection of PROXYL by stable amphiphilic nanostructures (ca. 23 nm) and a slow metabolic rate of macromolecules in this mCA. MRI signal contrast enhancement was achieved at the tumor site using the T1WI SE sequence conventionally used in clinical Gd^3+^-based CAs, while this mCA possessed great biosafety due to excellent biocompatibility and degradability of the DHPMA copolymer in this mCA. Therefore, Linear pDHPMA-mPEG-Ppa-PROXYL could have a great potential to act as a substitute of metal-based MRI CAs for clinical diagnosis of tumors.

## Supplementary Information

Additional file 1: **Scheme S1.** Preparation of Linear pDHPMA-mPEG-Ppa-PROXYL. **Fig. S1. **^1^H NMR spectra of Linear pDHPMA-SH a and Linear pDHPMA-mPEG-Ppa-PROXYL b (400 MHz, *d*_6_-DMSO as solvent). **Table S1.** Characterizations of polymers. **Fig. S2.** EPR spectrum of Linear pDHPMA-mPEG-Ppa-PROXYL. **Fig. S3.** Particle size of Linear pDHPMA-mPEG-Ppa-PROXY (ca. 23 nm, DLS). **Fig. S4. **TEM image of Linear pDHPMA-mPEG-Ppa-PROXYL. **Table S2.** Contents of amino acids in the polymer (wt%). **Fig. S5.** Zeta Potential of Linear pDHPMA-mPEG-Ppa-PROXYL (ca. 0 mV, DLS). **Fig. S6.** MR T1-weighted images of the liver a, kidney b and bladder c after 3-CP injection. **Fig. S7.** MR T1-weighted images of a tumor site after 3-CP injection. **Fig. S8. **A clinical Siemens 3.0 T MRI scanner was used to measure the longitudinal relaxivity (*r*_1_) of DTPA-Gd.

## Data Availability

All data generated or analyzed during this study are included in this published article and its Additional file 1.

## Abbreviations

MRI: Magnetic resonance imaging
CAs: Contrast agents
mCA: Macromolecular contrast agent
3-CP: 3-Carboxy-2,2,5,5-tetramethylpyrrolidine-1-oxyl
pDHPMA: Poly[*N*-(1, 3-dihydroxypropyl) methacrylamide
PEG: Polyethylene glycol
mPEG: Metholpolyethylene glycol
Ppa: Polyphthalamide-a
DLS: Dynamic light scattering
PBS: Phosphate buffer saline
TE: Echo time
TR: Repetition time
Fov: Field of view
EPR: Electronic paramagnetic resonance
SI: Signal intensity
CCK8: Cell Counting Kit-8
CLSM: Confocal laser scanning microscope
MW: Molecular weight
GFLG: Gly-Phe-Leu-Gly
PROXYL: 2,2,5,5-tetramethyl-1-pyrrolidinyl-*N*-oxyl
TEMPO: 2,2,6,6-tetramethylpiperidinyl-1-oxyl
RAFT: Reversible addition-fragmentation chain transfer
PTE: Pyridinyldithiol
ALT: Alanine aminotransferase
AST: Aspartate aminotransferase
CREA: Creatinine
