# Supplementary material for: A nitroxides-based macromolecular MRI contrast agent with an extraordinary longitudinal relaxivity for tumor imaging via clinical T1WI SE sequence
Source: J Nanobiotechnology. 2021 Aug 14;19:244. doi: 10.1186/s12951-021-00990-6 (PMC8364710; doi:10.1186/s12951-021-00990-6)
Supplement: Supplementary file 1 — Additional file 1: Scheme S1. Preparation of Linear pDHPMA-mPEG-Ppa-PROXYL. Fig. S1. 1H NMR spectra of Linear pDHPMA-SH a and Linear pDHPMA-mPEG-Ppa-PROXYL b (400 MHz, d6-DMSO as solvent). Table S1. Characterizations of polymers. Fig. S2. EPR spectrum of Linear pDHPMA-mPEG-Ppa-PROXYL. Fig. S3. Particle size of Linear pDHPMA-mPEG-Ppa-PROXY (ca. 23 nm, DLS). Fig. S4. TEM image of Linear pDHPMA-mPEG-Ppa-PROXYL. Table S2. Contents of amino acids in the polymer (wt%). Fig. S5. Zeta Potential of Linear pDHPMA-mPEG-Ppa-PROXYL (ca. 0 mV, DLS). Fig. S6. MR T1-weighted images of the liver a, kidney b and bladder c after 3-CP injection. Fig. S7. MR T1-weighted images of a tumor site after 3-CP injection. Fig. S8. A clinical Siemens 3.0 T MRI scanner was used to measure the longitudinal relaxivity (r1) of DTPA-Gd. [file 12951_2021_990_MOESM1_ESM.doc]

**Supporting Information**

*for*

**A nitroxides-based macromolecular MRI contrast agent with an extraordinary longitudinal relaxivity for tumor imaging *via* clinical T1WI SE sequence**

Shiwei Guo,^1,2,3,†^ Xiaoming Wang,^1,5,†^ Zhiqian Li,^1^ Dayi Pan,^1^ Yan Dai,^2^ Yun Ye,^2^ Xiaohe Tian,^1^ Hu Zhang,^1^ Zhongwei Gu,^1^ Qiyong Gong,^1,4^ and Kui Luo^1,4,*^

^1^Laboratory of Stem Cell Biology, and Huaxi MR Research Center (HMRRC), Department of Radiology, National Clinical Research Center for Geriatrics, Frontiers Science Center for Disease-Related Molecular Network, State Key Laboratory of Biotherapy, West China Hospital, Sichuan University, Chengdu, 610041, China

^2^Department of Pharmacy of the Affiliated Hospital of Southwest Medical University, Southwest Medical University, Luzhou, Sichuan Province, 646000, P. R. China

^3^Nuclear Medicine and Molecular Imaging Key Laboratory of Sichuan Province, Luzhou, 646000, P. R. China

^4^Functional and molecular imaging Key Laboratory of Sichuan Province, and Research Unit of Psychoradiology, Chinese Academy of Medical Sciences, Chengdu, 610041, China

^5^Department of Radiology, Chongqing General Hospital, University of Chinese Academy of Sciences (UCAS), No.104 Pipashan Main Street, Yuzhong District, Chongqing, 400014, China

^6^Amgen Bioprocessing Centre, Keck Graduate Institute, Claremont, CA 91711, USA

†Dr. Shiwei Guo and Xiaoming Wang contributed equally to this study.

*Corresponding author, E-mail: luokui@scu.edu.cn

**Materials and Methods**

All reagents used in this study were of HPLC grade. Starting materials including Linear pDHPMA-SH, Ppa-maleicimide and PTE-mPEG-PROXYL were prepared in the same approach we reported before [1, 2]. The weight-averaged molecular weight (MW) and polydispersity index (PDI) of polymers were measured *via* gel permeation chromatography (GPC) on an AKTA/FPLC system (GE Healthcare). The ^1^H NMR spectrum was recorded on a Bruker 400 (400 MHz) spectrometer. The Electron Paramagnetic Resonance (EPR) spectrum was acquired using a Bruker EMX plus X-band CW spectrometer. The size and zeta potential were detected by a Zetasizer (Malvern, Worcestershire, U.K.). The target copolymer was purified by fast protein liquid chromatography (FPLC) and characterized by size exclusion chromatography (SEC), ^1^H NMR analysis, amino acid analysis and dynamic light scattering (DLS). Mouse breast cancer cells (4T1) and human umbilical vein endothelial cells (HUVEC) were purchased from Chinese Academy of Sciences (Shanghai) Cell Bank. The CCK-8 kit was acquired from Dojindo (Japan). Female BALB/c mice were obtained from Chengdu DaShuo Biological Technology Co., Ltd. All animal experiments were strictly carried out in accordance with the Guidelines for Care and Use of Laboratory Animals of West China Hospital, Sichuan University and approved by the Animal Ethics Committee of China.

**Synthesis of** **Linear pDHPMA-mPEG-PROXYL**

At an ambient temperature, Linear pDHPMA-SH (100 mg, 0.26 mmol of sulfydryl) and DMF (5 mL) were added into a sealed flask and the mixture was stirred until Linear pDHPMA-SH was completely dissolved in DMF. After Ppa-maleimide (8 mg) was added into the above mixture, stirring continued for 5 h to give a green clear solution. PTE-mPEG-PROXYL (1.10 g, 0.43 mmol) and cat. AcOH (0.03 mL) were then added, and the reaction mixture was stirred for 2 days to give a light green clear solution. 50 mL of RO-water was slowly added to the reaction flask, followed by dialysis with RO-water as the medium for 24 h in the dark using a membrane (MWCO=8.0 kDa). After freeze-drying, Linear pDHPMA-mPEG-PROXYL was obtained as a light green solid (Weight: 330 mg, yield: 35%).

**Cellular uptake**

A confocal laser scanning microscope (CLSM) was used to observe uptake of Linear pDHPMA-mPEG-Ppa-PROXYL by 4T1 cells. 4T1 cells were seeded on 6-well glass plates at 5 × 10^4^ cells per well and incubated at 37 °C and 5% CO_2_ for 24 h. Linear pDHPMA-mPEG-Ppa-PROXYL was added to each well at a concentration of 0.5 μg/mL Ppa, and incubation continued for 1, 2 and 6 h. Cells were washed 3 times with PBS, and a nuclear dye Hoechst33342 reagent was added for 20 min incubation. Finally, fluorescence images of cells were acquired under the CLSM.

***In vitro* toxicity**

4T1 and HUVEC cells were selected to evaluate in vitro cytotoxicity of Linear pDHPMA-mPEG-Ppa-PROXYL. 4T1 and HUVEC cells were seeded onto 96-well plates at a density of 1 × 10^4^ cells/well. Linear pDHPMA-mPEG-Ppa-PROXYL at different concentrations (5 μg/mL, 2.5 μg/mL, 1.25 μg/mL, 0.625 μg/mL, 0.312 μg/mL, 0.156 μg/mL, 0.078 μg/mL and 0 μg/mL) was added to the modified eagle medium (MEM medium) to replace the initial cell culture media. Cells were further incubated for 24 h and washed three times with PBS after discarding the media. The cytotoxicity evaluation kit CCK-8 (Dojindo, Japan) was added to each well. After 2 h incubation in a cell incubator, the absorbance at 450 nm was read by a multifunctional enzyme labeler (Thermo Fisher SCIENTIFIC).

**Blood compatibility test**

**RBC hemolysis test**

The anticoagulant (containing anti-citrate sodium) was added into 2 mL of fresh blood from a healthy human body. The sample was centrifuged at 1000 g for 5 min, and washed 3 times with PBS. Finally, the upper supernatant was aspirated and red blood cells (RBCs) were re-suspended in PBS to prepare a 20% RBC suspension. The concentrations of Linear pDHPMA-mPEG-Ppa-PROXYL were set to 1 mg/mL, 2 mg/mL, and 5 mg/mL. PBS and deionized water were used as control groups. 1 mL of the above solution was transferred into an EP tube, followed by addition of 50 μL of RBC suspension. After incubation at 37 °C for 24 h, the samples were centrifuged (1000 g × 5 min). 200 μL of the supernatant was pipetted into a 96-well plate. The absorbance of the sample at 540 nm was read by a microplate reader. The above procedure was repeated three times. The percentage of the hemolysis rate was calculated by a method from a previous study [3].

**Morphology and aggregation of RBCs**

RBCs and the Linear pDHPMA-mPEG-Ppa-PROXYL solutions were prepared in the same manner as the above. After samples were incubated at 37 °C for 15-20 min and processed with centrifugation (1000 g × 5 min) to remove the supernatant, RBCs were fixed with 0.5 mL of 4% paraformaldehyde for 4 h. The fixed RBCs were re-suspended in PBS and 10-20 μL of the suspension was pipetted and evenly coated onto the bottom of a 24-well plate. The samples were successively dehydrated with 75%, 85%, 95% and 100% ethanol solutions. Finally, under a constant temperature of 25-30 °C, all samples were air dried, and stereotyped and sprayed with gold. The morphologies and aggregates of cells were observed under a scanning electron microscope (SEM).

***In vivo* toxicity**

Fifteen healthy female BALB/c mice (8-10 weeks, 20±2 g) were randomly divided into 3 groups (n = 5). These mice were injected with Linear pDHPMA-mPEG-Ppa-PROXYL and 3-CP at a dose of 0.135 mmol/kg PROXYL as well as saline (a control group) via tail vein, and all mice were sacrificed after 1 day. The blood samples of the mice were collected for blood chemistry index analysis and main organs (heart, liver, spleen, lung, kidney) were collected, fixed with a 4% paraformaldehyde solution for 48 h, and embedded in paraffin. Tissue sections were analyzed after hematoxylin-eosin staining.

**Results and Discussion**

**Scheme S1.** Preparation of Linear pDHPMA-mPEG-Ppa-PROXYL.

**Fig. S1.** ^1^H NMR spectra of Linear pDHPMA-SH **a** [1] and Linear pDHPMA-mPEG-Ppa-PROXYL **b** (400 MHz, *d_6_*-DMSO as solvent).

**Table S1.** Characterizations of polymers.

| Compounds | MW (kDa)^a^ | PDI^a^ | Spin conc.  (mmol/g)^b^ | Ppa  content^c^ | Ionic *r_1_*  (mM^−1^ s^−1^) ^d^ |
| --- | --- | --- | --- | --- | --- |
| Linear pDHPMA-SH ^[1]^ | 56 | 1.21 | / | / | / |
| Linear pDHPMA-mPEG-PROXYL | 102 | 1.22 | 0.111 | 0.64% | 0.93 |

^a^ The MW and PDI of polymers were measured *via* SEC (ÄKTA/FPLC system of GE Healthcare). ^b^ The spin conc. was expressed as mmol/g; The Ppa content as the weight percent (wt%). ^c^ The ionic relaxivity was defined as the relaxivity per nitroxides.

**Fig. S2.** EPR spectrum of Linear pDHPMA-mPEG-Ppa-PROXYL.

**Fig. S3.** Particle size of Linear pDHPMA-mPEG-Ppa-PROXY (ca. 23 nm, DLS).

**Fig. S4.** TEM image of Linear pDHPMA-mPEG-Ppa-PROXYL.

**Table S2.** Contents of amino acids in the polymer (wt%).

| Copolymer | Gly% | Phe% | Leu% | Lys% |
| --- | --- | --- | --- | --- |
| Linear pDHPMA-mPEG-PROXYL | 2.03 | 2.43 | 1.46 | 1.27 |

**Fig. S5.** Zeta Potential of Linear pDHPMA-mPEG-Ppa-PROXYL (ca. 0 mV, DLS).


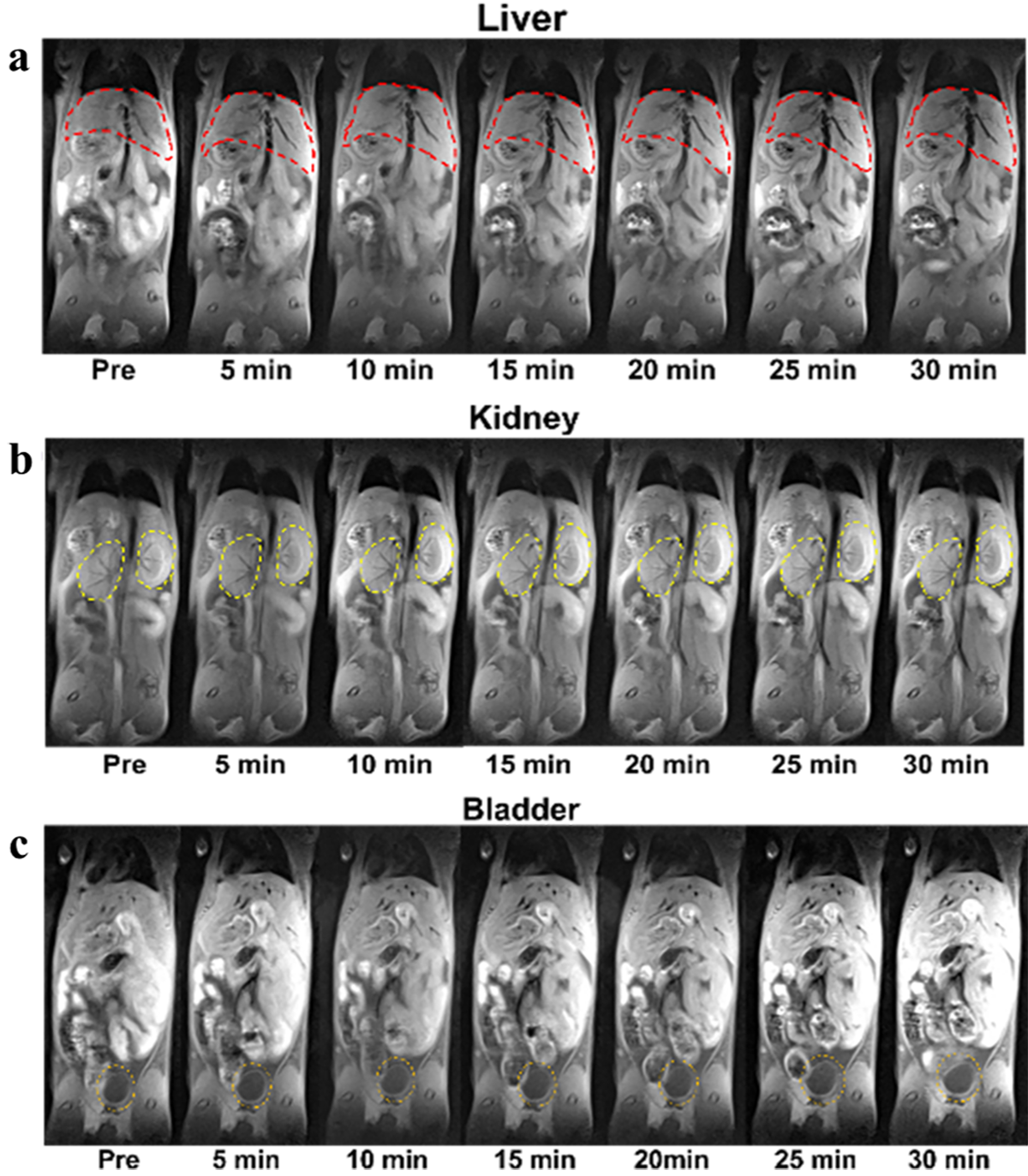


**Fig. S6.** MR T1-weighted images of the liver **a**, kidney **b** and bladder **c** after 3-CP injection.


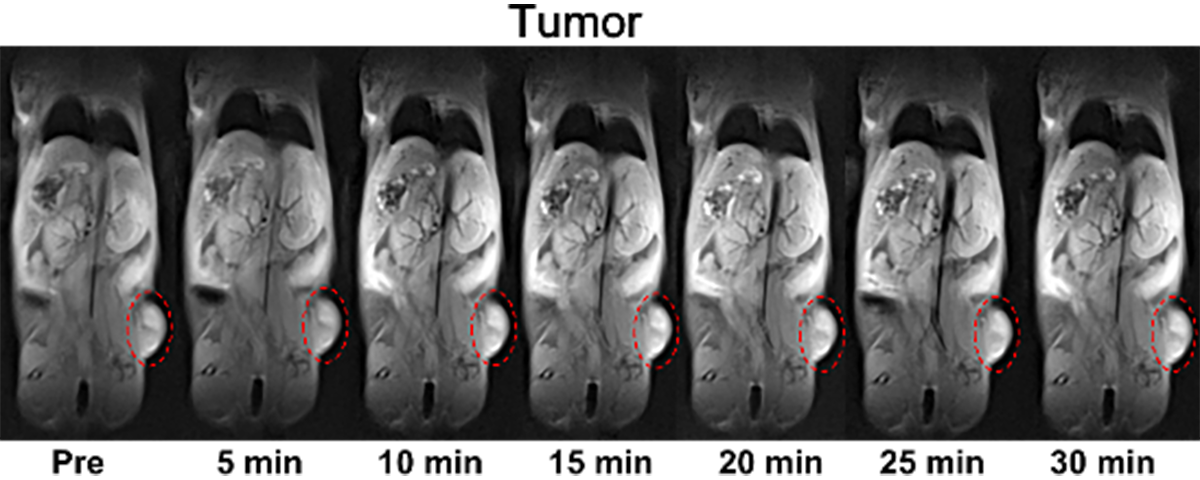


**Fig. S7.** MR T1-weighted images of a tumor site after 3-CP injection.


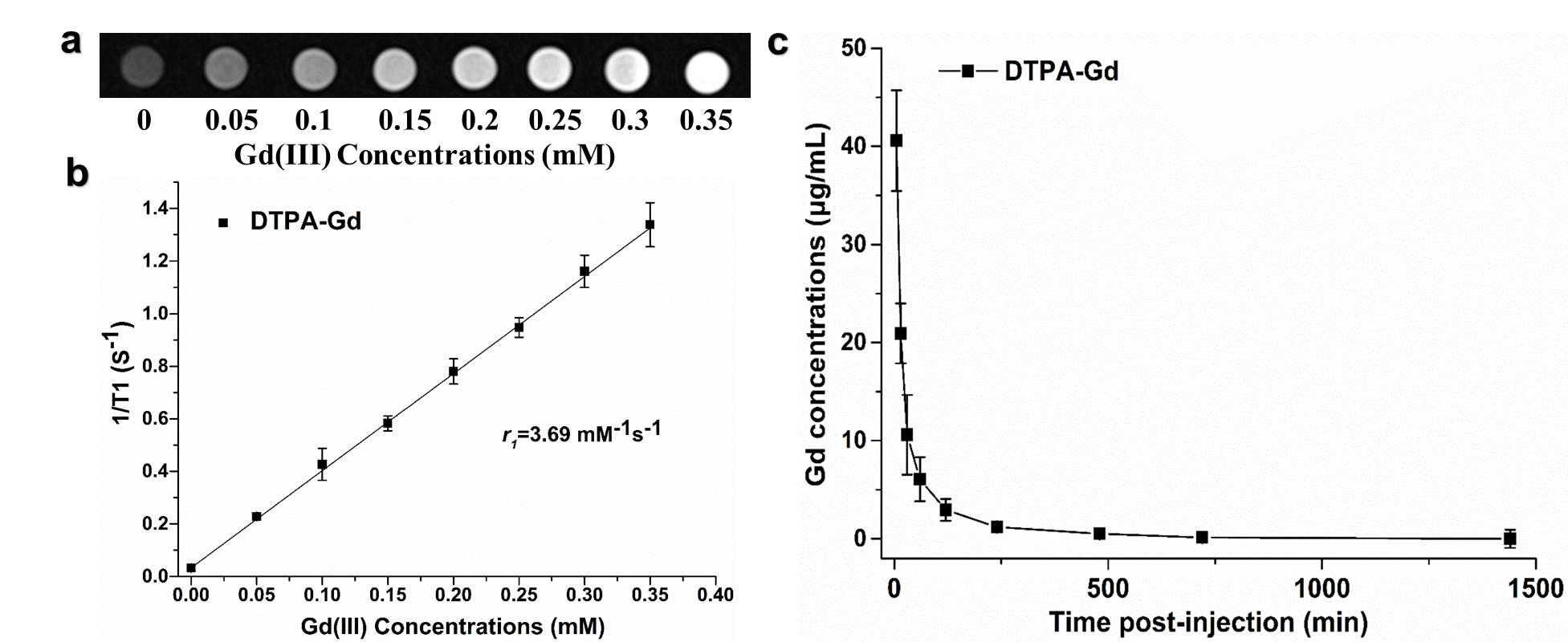


**Fig. S8.** A clinical Siemens 3.0 T MRI scanner was used to measure the longitudinal relaxivity (*r_1_*) of DTPA-Gd. Compared with Linear pDHPMA-mPEG-Ppa-PROXYL (**Fig. 2a**), MR signals for DTPA-Gd samples in Fig. S8a were more intense. The in vitro relaxivity (*r_1_* = 3.69 mM^-1^ s^-1^) of DTPA-Gd in Fig. S8b was significantly higher than that (*r_1_* = 0.93 mM^-1^ s^-1^) of Linear pDHPMA-mPEG-Ppa-PROXYL (**Fig. 2b**). This could be explained by that DTPA-Gd has seven unpaired electrons, which significantly increase the energy exchange with peripheral water molecules, resulting in a significant increase in the longitudinal relaxivity. However, only one electron is associated with Linear pDHPMA-mPEG-Ppa-PROXYL, therefore, it has a small longitudinal relaxivity. Although the in vitro relaxivity of Linear pDHPMA-mPEG-Ppa-PROXYL is weaker than that of DTPA-Gd, Linear pDHPMA-mPEG-Ppa-PROXYL as a non-metallic polymer has great biosafety, which may effectively avoid potential toxicity caused by DTPA-Gd as a metallic MR CA in clinical use. In addition, compared with Linear pDHPMA-mPEG-Ppa-PROXYL (**Fig. 3**), DTPA-Gd has a longer circulation time in vivo Fig. **S8c**, and the decline of its concentration in the blood is gradual and gadolinium is still detectable up to 12 h after injection. Because DTPA-Gd in the blood is not sensitive to the redox reaction as nitroxides to lose its relaxation effect, DTPA-Gd maintains blood circulation for a longer time.

**Reference**

1 Guo S, Xiao X, Wang X, Luo Q, Zhu H, Zhang H, Li H, Gong Q, Luo K. Reductive microenvironment responsive gadolinium-based polymers as potential safe MRI contrast agents. Biomater Sci. 2019;7:1919-1932.

2 Guo S, Wang X, Dai Y, Dai X, Li Z, Luo Q, Zheng X, Gu Z, Zhang H, Gong Q, Luo K. Enhancing the Efficacy of Metal-Free MRI Contrast Agents via Conjugating Nitroxides onto PEGylated Cross-Linked Poly(Carboxylate Ester). Adv Sci. 2020;7:2000467.

3 Li X, Sun L, Wei X, Luo Q, Cai H, Xiao X, Zhu H and Kui L. Stimuli-responsive biodegradable and gadolinium-based poly[*N*-(2-hydroxypropyl) methacrylamide] copolymers: their potential as targeting and safe magnetic resonance imaging probes. J Mater Chem B. 2017;5:2763-2774.
